# High-resolution T1 mapping with ANGIE detects increased right-ventricular extracellular volume fraction in patients with pulmonary arterial hypertension

**DOI:** 10.1186/1532-429X-17-S1-O39

**Published:** 2015-02-03

**Authors:** Bhairav B Mehta, Jorge A Gonzalez, Michael Salerno, Virginia K Workman, Sula Mazimba, Jamie L Kennedy, Elizabeth Gay, Kenneth C Bilchick, Frederick H Epstein

**Affiliations:** 1 Department of Biomedical Engineering, University of Virginia, Charlottesville, VA, USA; 2 Department of Radiology and Medical Imaging, University of Virginia, Charlottesville, VA, USA; 3 Medicine, University of Virginia, Charlottesville, VA, USA

## Background

Right ventricular (RV) failure is associated with increasing morbidity and mortality in pulmonary arterial hypertension (PAH)^1^. Pressure overload in PAH leads to a complex series of changes in cardiomyocytes and the extracellular matrix of the RV^2^. Thus, quantitative T1 mapping of the RV to assess myocardial extracellular volume fraction (ECV) may be a valuable noninvasive marker of RV fibrosis in these patients. However, current clinically preferred T1 mapping techniques have limited spatial resolution, restricting their application to assessment of left ventricular (LV) ECV. We recently developed a novel technique, termed ANGIE^3^, which uses navigator gating and acceleration with compressed sensing (CS) to provide high-resolution T1 mapping for assessment of thin structures such as the wall of the RV. The aim of the present study was to use ANGIE to test the hypothesis that RV ECV is elevated in patients with PAH compared to reference subjects.

## Methods

Eighteen patients were recruited to undergo cardiac magnetic resonance imaging (CMR) with gadolinium on a 1.5T system (Avanto Siemens) -- 10 patients (60±16.6 yrs) with PAH and 8 patients with chronic left ventricular systolic heart failure (LHF) (60.5±7.6 yrs). The MRI protocol included: 1) SSFP cine imaging, 2) pre-contrast high-resolution ANGIE T1 mapping (1.2-1.5x1.2-1.5x4mm^3^), 3) late gadolinium enhancement (LGE) acquisitions at 10 mins after gadolinium injection (0.15mmol/kg), and 4) multiple post-contrast ANGIE acquisitions at intervals of 5 mins up to 30 mins post-contrast. Additionally, modified Look-Locker inversion recovery (MOLLI)^4^ scans were performed along with ANGIE in 8 out of the 10 PAH patients for validation of ANGIE LV ECV measurements. 15 patients (10 with PAH, 5 with LHF) had enough tricuspid regurgitation by echocardiography to measure RV systolic pressure (RVSP).^5^

## Results

**Figure 1:**
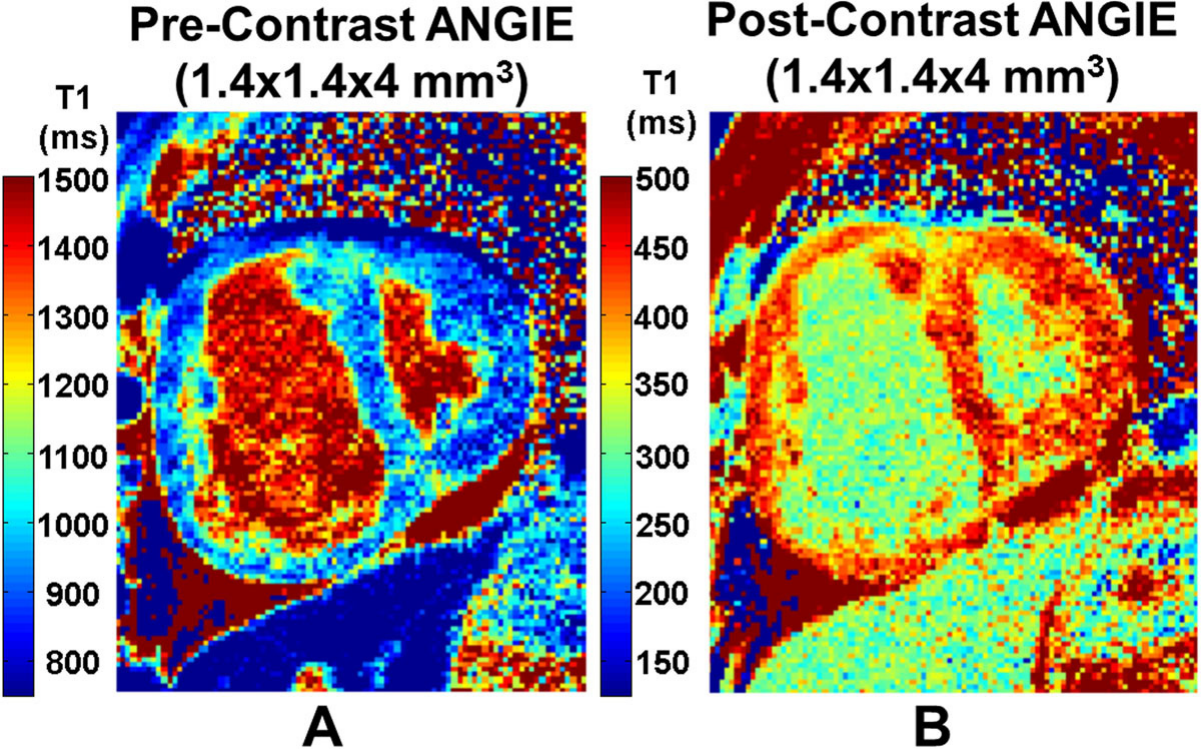
Example ANGIE T1 maps acquired from a patient with pulmonary arterial hypertension (PAH). A: T1 map prior to injection of gadolinium. B: ANGIE T1 map 25 minutes after injection of gadolinium. ANGIE T1 maps show good definition of the RV wall.

**Figure 2:**
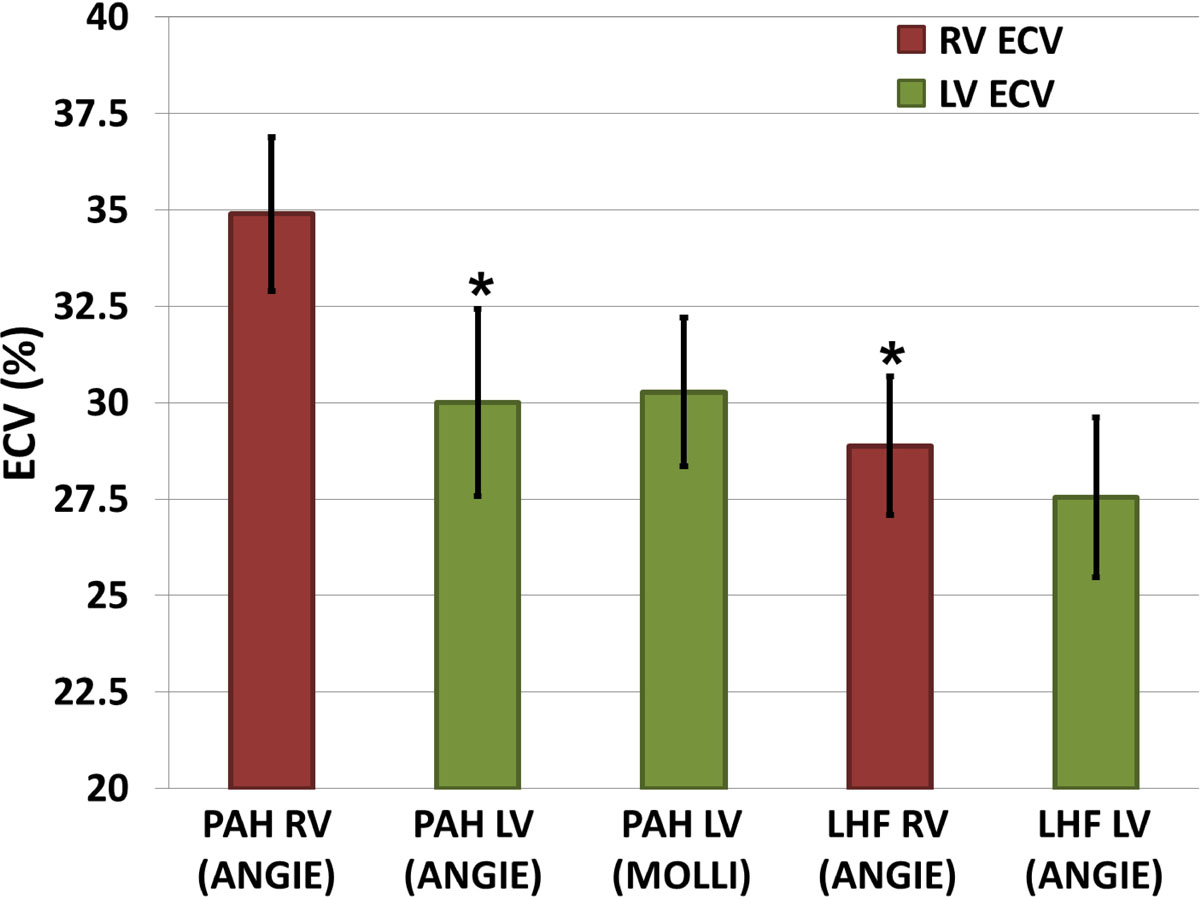
Myocardial extracellular volume fraction (ECV) measurement results in patients with pulmonary arterial hypertension (PAH) and chronic left ventricular systolic heart failure (LHF). RV ECV is significantly higher in PAH versus LHF groups (p<0.01). In addition, RV ECV is significantly higher than LV ECV in PAH (p<0.01), but RV ECV and LV ECV are similar in LHF. *: p<0.01 vs. PAH RV (ANGIE).

Figure 1 illustrates high-resolution pre- and post-contrast T1 maps of the LV and RV acquired from a PAH patient using ANGIE. The ANGIE measurements of LV ECV (in regions excluding scar on LGE) in PAH patients were in close agreement with MOLLI (Figure 2), confirming the accuracy of ECV measurements performed using ANGIE. Greater RV ECV by ANGIE was correlated with increasing RV systolic pressure (R =0.560, p<0.05) and decreasing RV ejection fraction by CMR (R =0.564, p<0.02). RV ECV in PAH was also significantly greater than RV ECV in LHF (p<0.01) (Figure 2). In addition, the RV ECV was also significantly greater than the LV ECV in PAH patients (p<0.01), but RV ECV and LV ECV were similar in LHF patients (p=NS) (Figure 2).

## Conclusions

Pre- and post-contrast ANGIE imaging provides high-resolution T1 mapping and ECV assessments for not only the LV but also the thin-walled RV, with LV ECV by ANGIE and MOLLI in close agreement. The extent of RV fibrosis in PAH, as measured by RV ECV, increases with RV pressure overload and decreasing RV systolic function. RV ECV is greater than LV ECV in PAH and similar to LV ECV in LHF.

## Funding

This work was funded by Siemens Medical Solutions, NIH R01 EB 001763, NHLBI K23 HL 094761, NHLBI K23 HL 112910, NIH T32 EB 003841, AHA Grant-in-Aid 12GRNT12050301, and AHA Predoctoral Award 14PRE20210008.
